# Lacrimal gland organoids: A systematic review on development, characterization, molecular profiling and translational potential in dry eye disease

**DOI:** 10.1016/j.exer.2026.110956

**Published:** 2026-03-03

**Authors:** Mohammad Gufran Siddiqui, Vanessa L. LaPointe, Mor M. Dickman, Sayan Basu, Vivek Singh, Swati Singh

**Affiliations:** a Brien Holden Eye Research Center (BHERC), LV Prasad Eye Institute, Hyderabad, Telangana, India; b Shantilal Shanghvi Cornea Institute, LV Prasad Eye Institute, Hyderabad, Telangana, India; c Hariram Motumal Nasta & Renu Hariram Nasta Ophthalmic Plastic Surgery Services, KAR Campus, LV Prasad Eye Institute, Hyderabad, Telangana, India; d Prof. Krothapalli Ravindranath Ophthalmic Research Biorepository, LV Prasad Eye Institute, Hyderabad, Telangana, India; e MERLN Institute for Technology-Inspired Regenerative Medicine, Maastricht University, Maastricht, the Netherlands; f Department of Ophthalmology, Division of Surgical Specialties, Utrecht University Medical Center, Regenerative Medicine Center Utrecht (RMCU), Utrecht University, Utrecht, the Netherlands

**Keywords:** Lacrimal gland, Organoid, Stem cells, PAX6, Dry eye disease, Aquaporin

## Abstract

Organoids are mini-organs engineered to mimic the native tissue’s organization, cellular structure, and function. Lacrimal gland organoids are considered a potential treatment for patients with dry eye, but the gland’s complex heterogeneity has been difficult to replicate. This systematic review summarizes methods for creating lacrimal gland organoids, their characterization, and potential applications. Data collected included organoid source, composition of expansion or differentiation media, biomarkers, gene expression, responses to stimulants, and effects in animal models. The sources of lacrimal gland organoids were human induced pluripotent stem (hiPS) cell lines (n = 2) and tissue biopsies from humans, mice, or pigs (n = 5). Tissue-derived organoids from mice grew for 40 passages, while those from human biopsies lasted up to 20 passages. There is a need to optimize the culture protocol to preserve cell composition and support long-term growth. The organoids expressed epithelial markers (KRT5, KRT13, AQP), mesenchymal markers (Vimentin and α-SMA), and developmental markers (PAX6, TP63, and OCT3/4), though cellular proportions varied between studies. Stimulation studies showed increased calcium influx and β-glucosaminidase activity, indicating secretory capacity. RNA sequencing revealed unique gene expression patterns associated with stemness and functional maturity, including tear proteins and markers of ductal and myoepithelial cells. PAX6 knockout studies confirmed PAX6’s essential role in organoid growth. Published studies lack data on epithelial polarity, the coexistence of ductal and acinar cells within organoids, and the in vivo secretory function of organoids. Transplanted organoids into animal models of dry eye disease (two immunosuppressed and two naïve) remained viable for 8 weeks and expressed tear-related markers (AQP5, KRT14, PAX6), although there was no data on tear film or ocular surface changes. Future research could explore the effects of transplantation on the ocular surface and host immune responses.

## 1 Introduction

The main lacrimal gland (LG) is a seromucous, acinotubular merocrine gland located in the upper outer part of the orbit (Singh & Basu., 2020; Singh et al., 2024). The gland lies obliquely between the orbital bones and soft tissue, extending from the superior rectus muscle to the frontozygomatic suture (Singh & Basu., 2020; Singh et al., 2023). It is divided into two lobes: the larger orbital lobe, which comprises 60-70%, and the smaller palpebral lobe, with a composition of mainly acinar, ductal, and myoepithelial cells in the lobes along with lymphocytes, plasma cells, fibroblasts, blood vessels, and nerve fibers (Singh & Basu., 2020; Singh et al., 2023). Acinar and ductal cells are crucial for aqueous secretions of the tear film, while myoepithelial cells facilitate secretion by applying pressure to the acini (Singh & Basu., 2020; Singh et al., 2023, Fig. 1).

Lacrimal gland dysfunction, resulting from injuries, infections, autoimmune diseases like Sjögren’s syndrome, or age-related degeneration, results in aqueous-deficient dry eye (ADDE) (Bron et al., 2019; Donthineni et al., 2023). This condition is marked by tear hyperosmolarity and instability of the ocular surface, contributing to the multifactorial dry eye disease, which can vary from mild to chronic forms (Craig et al., 2017; Jones et al., 2017). Current treatments for ADDE include artificial tears, anti-inflammatory medications (e.g., cyclosporine A), punctal occlusion, tear stimulants (Donthineni et al., 2023; Craig et al., 2017; Jones et al., 2017), autologous serum (Pan et al., 2017), punctal occlusion (Ranjan et al., 2024), and neuro-stimulation devices to enhance tear production (Yu et al., 2021). These therapies are temporary and offer only symptomatic relief without addressing the root cause. Due to the complexity of lacrimal gland dysfunction, new approaches are being explored, including stem cell therapy, nanotechnology, and hydrogels (Chen et al., 2025; Joshi et al., 2023; Williams and Mann, 2014; Han et al., 2021). Stem cell therapy shows promise for regenerating damaged lacrimal gland tissue, demonstrated by the transplantation of mesenchymal stem cells (Joshi et al., 2023; Veernala et al., 2022; Møller-Hansen et al., 2024; Dietrich et al., 2016) and iPSCs (Dietrich et al., 2016) at the target site. Studies on human and mouse lacrimal glands have confirmed the presence of stem cells within the glands, which can be grown in vitro. These culture experiments aim for two main outcomes: developing in vitro lacrimal gland models that mimic native tissue, and creating a 3D, transferable lacrimal gland cell product capable of regenerating the native gland or simulating its function. Traditional 2D culture systems (Oliver, 1980; Hann et al., 1989; Veernala et al., 2022) have long been used to study lacrimal gland function and disease mechanisms, including producing immortalized lacrimal gland cell lines (Nguyen et al., 1999; Gleixner et al., 2024); however, they fail to replicate the complex microenvironment and interactions found in vivo. For developing a 3D lacrimal gland, cultures have successfully been grown in microgravity conditions with up to 28 days of viability (Schrader et al., 2009). The 2D culture lacks the complex three-dimensional organization of the acinar, ductal, and stromal components of the lacrimal gland. Lacrimal gland spheroids, or “lacrispheres,” have been developed as promising tools for gland regeneration but have not yet been tested in animal models (Massie et al., 2018; Tiwari et al., 2018). Lacrispheres differ from organoids as they are simple cell aggregates; organoids are more complex. Organoids are three-dimensional mini-structures derived from stem cells that replicate the architecture and function of an organ.

There are many uncertainties in defining lacrimal gland organoids, including their specific cellular composition—whether they contain ductal or acinar cells, the presence of a ductular system rather than just ductal cells, and the proportions of neural and mesenchymal cells. Organoids are created from adult tissue-specific stem cells (ATSS), embryonic stem cells (ESCs), or induced pluripotent stem cells (iPSCs), which differentiate into various cell types representing ocular lineages such as the cornea, retina, lens, and conjunctiva (Qiu et al., 2012; Erbani et al., 2016; Foster et al., 2017; Zhang et al., 2017; Chakrabarty et al., 2018; Higa et al., 2020; Hongisto et al., 2018, Manafi et al., 2021; Kasal et al., 2022; Bannier-Hélaoüet et al., 2024). Successfully generated exocrine gland organoids (Zhao et al., 2021; Yoon et al., 2024; Grapin-Botton and Kim, 2022), including lacrimal gland organoids (Grapin-Botton and Kim, 2022; Hayashi et al., 2022; Asal et al., 2023; Jeong et al., 2021; Bannier-Hélaoüet et al., 2021; Bannier-Hélaoüet et al., 2023; Rodboon et al., 2022a,b; Hayashi et al., 2016; Li et al., 2019; Rodboon et al., 2022), have been developed to replicate their structural and functional features. This review focuses on techniques for creating lacrimal gland organoids using iPS cell lines and tissue samples, their characterization, composition, functional assays, and a critical evaluation of their potential for translation in animal models.

## 2 Methods

A systematic review of lacrimal gland organoids was conducted in accordance with PRISMA guidelines. Search terms used on PubMed and Scopus were “lacrimal gland culture”, lacrimal gland organoid”, and “lacrimal gland 3D cultures”. Original research articles were included that investigated lacrimal gland organoids using animal or human tissues or induced pluripotent stem (iPS) cell lines. The abstracts of 45 articles were screened by two authors (M.G.S., S.S.), and seven were selected, focusing on lacrimal gland organoids. Following screening, data were independently extracted by two authors (S.S, M.G.S), including study details such as author(s), title, organoid source, digestion or differentiation method (for iPS cell lines), the matrix used, expansion or differentiation media, the number of passages or differentiation days (for iPS cell lines), biomarkers analyzed via immunofluorescence (IF), immunohistochemistry (IHC), western blot, and enzyme-linked immunosorbent assay (ELISA), gene expression assessed using quantitative reverse-transcriptase polymerase chain reaction (qRT-PCR), stimulants used in functional assays, as well as metabolomics, proteomics studies, and RNA sequencing (bulk or single-cell).

**Fig. 1:**
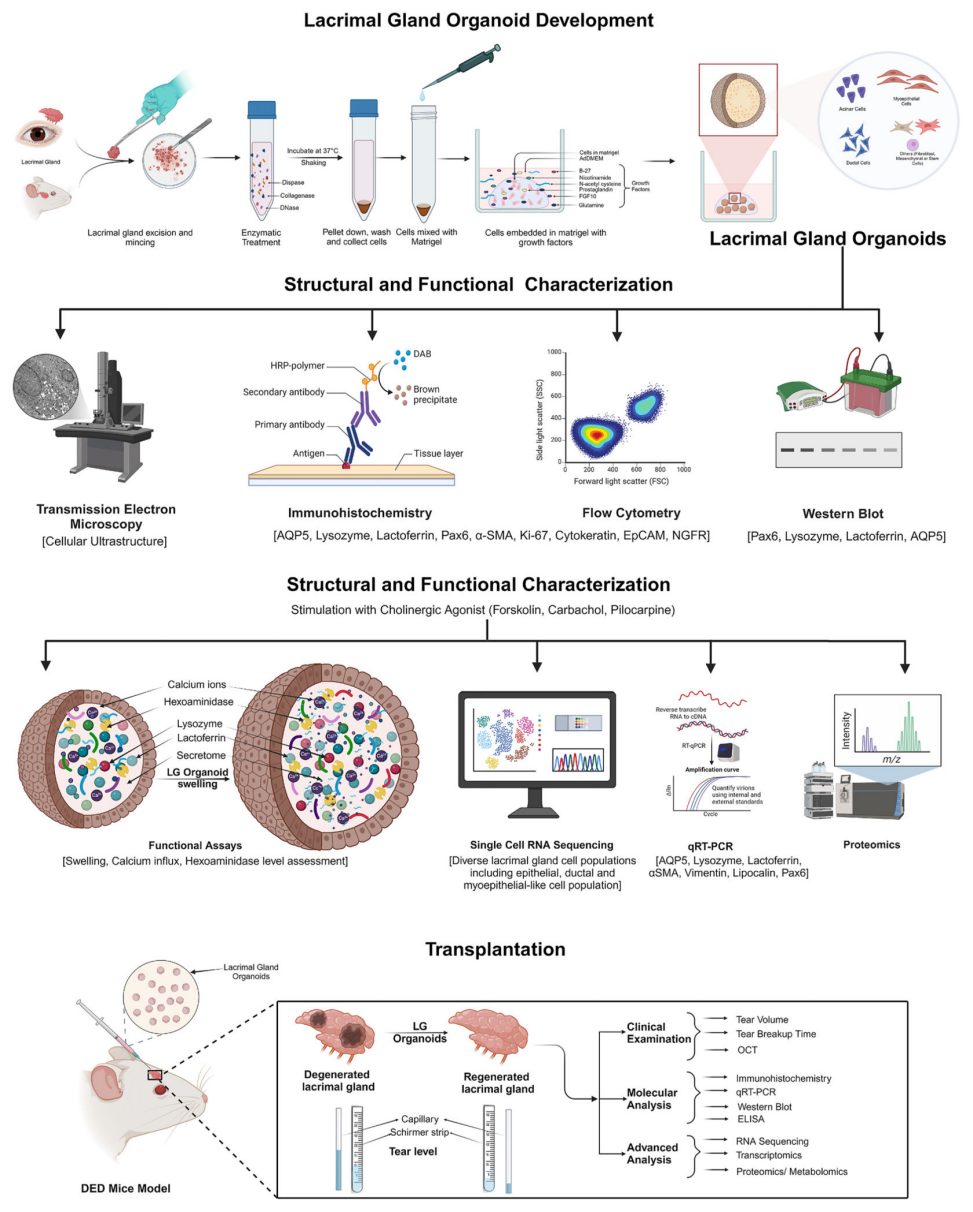
Schematic showing the steps involved in lacrimal gland organoid development, characterization, and translational uses.

Additionally, the animal model used, organoid delivery methods, and specific outcome measures related to functional assessments or gene expression were documented. All extracted data were compiled in MS Excel (Version 2409) for analysis and comparison across studies. Fig. 1 illustrates the comprehensive workflow of lacrimal gland organoid development, followed by structural and functional characterization and translational use.

## 3 Overview of lacrimal gland organoid studies

### 3.1 Organoid tissue source

Of the six studies, two used human-induced pluripotent stem cell lines (Hayashi et al., 2022; Asal et al., 2023), and five used lacrimal gland biopsies (Jeong et al., 2021; Bannier-Hélaoüet et al., 2021; Bannier-Hélaoüet et al., 2023; Rodboon et al., 2022a,b; Nguyen et al., 2025). The lacrimal gland was obtained from humans and mice in four studies (Jeong et al., 2021; Bannier-Hélaoüet et al., 2021; Bannier-Hélaoüet et al., 2023; Nguyen et al., 2025), and one study used porcine tissue (Rodboon et al., 2022a,b). The hiPS cell lines used were generated by Akbari et al., 2019 for one study (Asal et al., 2023), and 201B7 & human ES cell lines (KhES-1) were used by the other (Hayashi et al., 2022). The details are summarized in Table 1. Patients from whom biopsies were taken had no LG disorder. The size of the tissue biopsy was either the entire lacrimal gland or a partial lacrimal gland. One of the studies involving human samples describes a protocol-based approach, without any specific mention of the tissue source (Bannier-Hélaoüet et al., 2023).

### 3.2 Expansion and differentiation of LG organoids using iPS cell lines

Two studies employed different methodologies for generating lacrimal gland organoids from induced pluripotent stem cells (iPSCs). iPSCs are directed towards the neuroectodermal lineage, and PAX6 and SOX9-positive ectodermal cells are sorted, and the addition of differentiation media supports the lacrimal gland organoid formation. The variations were in the media composition and the time required for organoid differentiation (Table 1). The specific role of the factors added to the expansion or differentiation media is listed in Table 2. Asal et al. employed a multi-stage protocol, transitioning from mTeSR1 medium to Eye Field and Ocular Differentiation media. This approach mimicked epithelial-mesenchymal interactions, driven by FGF10 and BMP7 signaling, essential for glandular budding and branching. This method generated lacrimal gland organoids in approximately seven weeks. Conversely, Hayashi et al. implemented a phased differentiation protocol, initiating with StemFit medium for ten days. The development of lacrimal gland-like organoids from SEAM-derived clusters utilized key signaling pathways essential for glandular differentiation. FGF10 and BMP7 supported the process by mimicking epithelial-mesenchymal interactions. Organoids started to form by day 4 in Matrigel, supported by extracellular matrix signaling. Organoids matured into three-dimensional structures in six weeks. Although there were minor differences in differentiation duration and media used, both methodologies target the FGF and BMP pathways for branching and mesenchymal-epithelial interactions, effectively producing lacrimal gland organoids.

### 3.3 Convergent signaling pathways

Lacrimal gland development involves epithelial-mesenchymal interactions and epithelial budding and branching. There are multiple mechanisms involved in lacrimal gland development; however, the signalling pathway involved in organoid development has not been studied in detail. FGF signaling represents the central inductive pathway governing lacrimal gland development. During embryogenesis, FGF10 is secreted by periocular mesenchyme and acts on the lacrimal gland epithelium to initiate epithelial budding and gland formation; genetic ablation of Fgf10 results in the complete absence of lacrimal gland development (Govindarajan et al., 2000; Makarenkova et al., 2000). Based on these developmental findings, FGF10 was included in the expansion and differentiation media of organoid development protocols from adult tissues and iPSCs. Activation of its receptor, FGFR2b, on epithelial progenitors supports branching morphogenesis and epithelial expansion (Finburgh et al., 2023; Jones et al., 2019; Pan et al., 2008). FGF signaling does not act in isolation but interacts with other morphogenetic pathways, particularly BMP, Wnt, and Notch signaling, to regulate epithelial fate decisions and branching morphogenesis. BMP signaling, particularly via BMP7, forms mesenchymal condensations around epithelial buds, promoting branching and epithelial differentiation (Dean et al., 2004). Consistent with this developmental role, BMP7 was incorporated into the iPSC-derived organoid protocol (Asal et al., 2023) to recapitulate early ocular lineage cues in vitro. In contrast, adult tissue-derived organoid systems did not supplement BMP ligands but instead modulated BMP signaling indirectly by including the BMP antagonist Noggin, as unchecked BMP signaling can dysregulate mesenchymal cell proliferation and hinder the maintenance of epithelial progenitor populations.

Wnt signaling plays a dual role in lacrimal gland development, supporting the maintenance of epithelial progenitors under normal conditions while preventing excessive epithelial budding. Developmental studies have shown that elevated canonical Wnt activity can suppress FGF10-mediated lacrimal gland morphogenesis and BMP7-induced mesenchymal proliferation (Dean et al., 2005; Patel et al., 2011). Wnt signaling is crucial for the in vitro expansion of mouse acinar cells in organoids, and Wnt-mimetics have been shown to restore acinar cells in a mouse model of duct ligation (Nguyen et al., 2025). In organoid culture, this balance is achieved by the controlled use of the Wnt agonist R-spondin 3, which enhances canonical Wnt signaling during early expansion phases to maintain progenitor populations and is then removed during differentiation to allow epithelial maturation and branching. This method mimics gland morphogenesis, in which regulation of Wnt signaling is essential for the transition from progenitor expansion to epithelial differentiation. In contrast, acinar organoid systems require sustained Wnt pathway activation to maintain acinar identity and proliferative capacity (Nguyen et al., 2025).

Notch signaling regulates epithelial organization and differentiation during epithelial branching morphogenesis (Dvoriantchikova et al., 2017). Studies of salivary gland development indicate that Notch signaling influences epithelial growth and differentiation via γ-secreta-se-dependent mechanisms (Dang et al., 2009). Organoid culture media use the γ-secretase inhibitor DAPT to regulate Notch signaling during differentiation, thereby promoting epithelial maturation, decreasing proliferation, and promoting lineage commitment to tear-producing epithelial cells. However, Notch 1 signaling in the mouse lacrimal gland induced conversion of basal cells into luminal epithelial cells in proliferating cells and not in the resting state. However, despite its known role in epithelial differentiation, the effects of Notch on lumen formation within organoids remain unclear, as does the precise contribution of Notch signaling to acinar versus ductal specification.

**Fig. 2:**
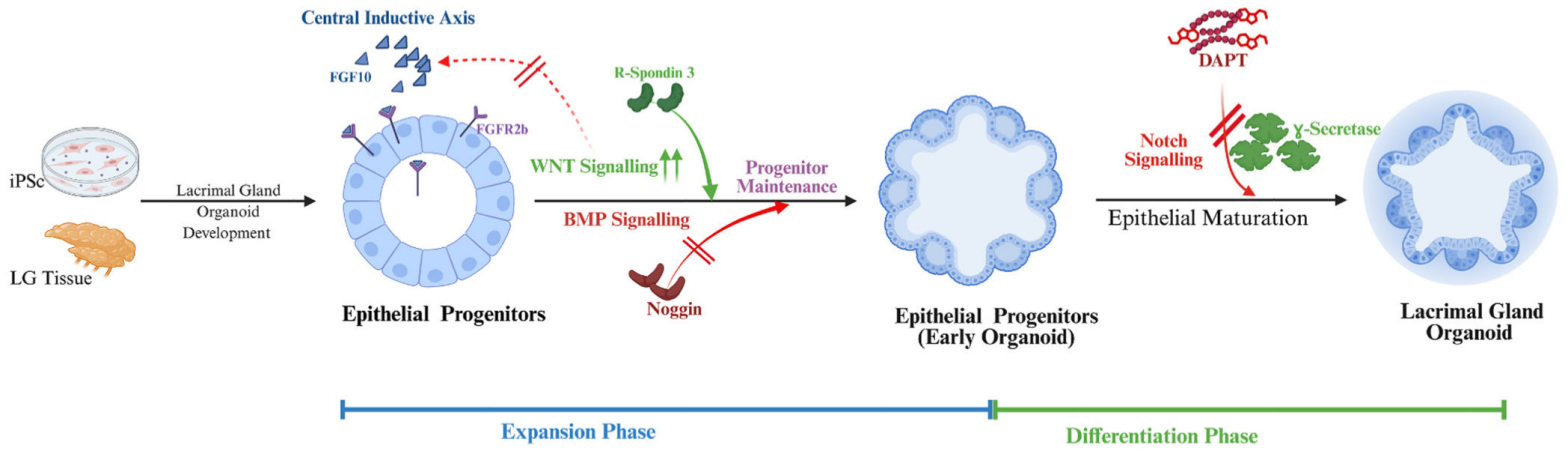
Convergent developmental signaling pathways regulating lacrimal gland organoid formation. Different key developmental signaling pathways converge on the FGF10–FGFR2b axis as the central inductive driver of epithelial budding and branching morphogenesis. BMP signaling, modulated by BMP7 inhibition via Noggin, maintains epithelial progenitors. Canonical Wnt signaling, potentiated by R-spondin-3 during early culture phases, supports epithelial progenitor maintenance. Notch signaling, modulated through γ-secretase inhibition using DAPT, influences epithelial maturation during later stages of organoid differentiation.

The likely unified mechanistic signaling pathway in lacrimal gland organoid development includes FGF10-induced epithelial budding in expansion medium, supported by Wnt and BMP7, followed by Notch inhibition during differentiation (Table 3 & Fig. 2). Expansion media contain the factors present during in vivo lacrimal gland development, branching, and epithelial proliferation. Across all studies, FGF10 was used to develop lacrimal gland organoids. hiPSC protocols emphasize FGF10/BMP7; ATSS protocols add a ROCK inhibitor (Y-27632) to improve viability and a WNT agonist (R-spondin 3).

### 3.4 Expansion and differentiation of LG organoids using tissue biopsy

Tissue biopsies were divided into a 1 mm size before digestion. Different studies have explored different digestion protocols. (Jeong et al., 2021; Bannier-Hélaoüet et al., 2021, 2023) (Table 1). Jeong et al. (2021) implemented a digestion protocol utilizing dispase II, DNase I, and collagenase II, along with a specialized expansion medium derived from salivary gland organoid culture, which allowed organoid growth for up to 19 passages. In contrast, Bannier-Hélaoüet et al. (2023) applied a more concentrated collagenase I digestion method and a comprehensive mix of expansion media that included various growth factors and conditioned media, resulting in mouse organoids that proliferated within 7-10 days and differentiated in 5 days. The human LG organoids from the same utilized a similar media composition but removed prostaglandin E2 (PGE2). Bannier-Hélaoüet et al. (2021) also employed collagenase in conjunction with the ROCK inhibitor Y-27632, which improved cell viability. Their expansion media for mouse organoids incorporated a wide array of factors, while the media for human organoids contained elevated levels of Noggin and R-spondin 3. Importantly, their mouse organoids could be passaged up to 40 times. In contrast, human organoids reached at least 20 passages, indicating a superior long-term culture capability compared to the 19 passages achieved by Jeong et al. (2021). Nguyen et al. (2025) generated acinar cell-derived lacrimal gland organoids from adult murine tissue using enzymatic digestion and culture in Wnt mimetic-supplemented medium containing EGF and FGFs, enabling sustained acinar organoid expansion and passaging. The study conducted by Rodboon et al., in 2022 used magnetic three-dimensional bioprinting (M3DB) to generate organoids, in which cells were magnetized with a specific volume of magnetic nanoparticle solution (MNP). The media used was Epithelial Enrichment Media (EEM), which led to an increase in organoid size from 173 ± 17.64 μm to 628 ± 24.26 μm over 8 days of culture.

### 3.5 Organoid characterization

Different biomarkers were studied using techniques such as IF, Western blot, flow cytometry, ELISA, and qRT-PCR across the studies to characterize the organoids. The details are listed in Table 4. Besides the markers listed in the table, the structural proteins, including α-crystallin, Lamin A + C, and β-Actin, have been studied by Hayashi et al. (2022). The ion pump/channels, Na+/K + ATPase, and Pannexin 1 were analyzed by Asal et al. (2023) for the secretory role of the organoids.

#### 3.5.1 RNA sequencing

Two studies sequenced organoids’ RNA using bulk and single-cell RNA sequencing. Bulk mRNA sequencing highlighted distinct gene expression in lacrimal gland organoids cultured in expansion medium (EM) and differentiation medium (DM). Organoids in EM showed upregulation of stem cell markers, including Lgr5 and Axin 2. At the same time, those in DM expressed markers of myoepithelial cells (Acta2, Myl9) and proteins associated with tear production. Markers Acta2 and Myl9 are also expressed in mural cells of mesodermal origin, such as vascular smooth muscle cells. Additionally, DM promoted immune defense genes and retinol metabolism, suggesting functional maturation. Single-cell RNA sequencing (scRNA-seq) revealed diverse lacrimal gland cell populations, including 800/916 epithelial cells producing tear proteins (LTF, LCN1, LYZ, AQP5, FOXC1), 16 myoepithelial cells (ACTA2, MYH11, MYLK, and TPM2), 11 endothelial cells (CDH5, PECAM1), and 90 lymphocytes (CD2) (Bannier-Hélaoüet et al., 2021). Organoids in EM resembled basal ductal cells, whereas DM enhanced tear-related proteins such as AQP5 and MUC16, thereby validating their role as functional models (Bannier-Hélaoüet et al., 2021). Hayashi et al. (2022) found that by day 10 of 3D culture, lacrimal gland progenitors expressed proliferation markers (MKI67) and epithelial-mesenchymal transition markers (VIM, FN1), which declined by day 20. By then, AQP5+ acinar cells produced functional proteins such as lipocalin (LCN2) and defensin (DEFB1), and organoids contained acinar, ductal, and myoepithelial-like cells. Specific myoepithelial cell markers, calponin, FOXC1, CK14, and alpha-SMA, were positive on staining in one study only, at day 30-35 of differentiation (Asal et al., 2023). The cellular composition of organoids is heterogeneous across studies, and the replicability of cell proportions across passages and media needs further study.

#### 3.5.2 Distinction between acinar and ductal cells

A persistent problem in 2D or 3D lacrimal gland culture systems is correctly identifying acinar cells from ductal epithelial cells. Both are of ectodermal lineage and have a secretory phenotype (Farmer et al., 2017). In a normal lacrimal gland, the pyramidal shape of acinar epithelial cells, arrangement in clusters, and presence of myoepithelial cells around them differentiate from ductular epithelial cells that have a cuboidal to columnar shape and are arranged in two layers of cells, basal and luminal columnar cells Farmer et al. (2017); M.C. Edman et al., 2010; Asal et al. (2023) reported epithelial cells in organoids could be acinar or ductal based on positive LCN2, CK19, PANX1, and AQP5 expression. However, they suggested that PANX1 and CK19 are luminal ductal epithelial cell markers that support ductal predominance. CK19 is also expressed in acinar epithelial cells. However, CK15, a progenitor cell marker, is expressed in basal ductal epithelial cells, which were positive in their organoids. Jeong et al. (2021) supported the idea that organoids are acinar mainly due to the expression of SMA positivity around the epithelial cells. However, no RNA expression data were available for these organoids. This contrasted with another study, in which organoids were predominantly labeled as ductal in origin (Bannier-Hélaoüet et al., 2021). Single-cell RNA sequencing revealed a population of ductal cells expressing LCN2 and WFDC2; however, an IHC image in their paper shows LCN2 positivity in acinar cells as well (Bannier-Hélaoüet et al., 2021). Another reason to assume the ductal nature of organoids was the absence of MIST-1, an acinar cell marker, in the organoids, which confirmed the ductal nature of the organoid (as per personal communications with author S.S). In their study, CK5-expressing cells, labeled as ductal progenitor cells, were present in organoids cultured in expansion medium and progressed to LCN2- and WFDC2-positive duct-like cells in differentiation medium. It remains unclear whether the organoids contain a mixed population of acinar and ductal epithelial cells. Also, the factors that contribute to the differentiation of epithelial cells into acinar or ductal epithelial cells in the organoid culture are unclear. In the developing mouse lacrimal gland, three cell lineages are observed: epithelial, mesenchymal, and immune (Farmer et al., 2017). Epithelial lineage committed cells give rise to acinar, ductal, and myoepithelial cells. In the postnatal stage, the acinar epithelial and myoepithelial cells are closely apposed, whereas ductal epithelial cells, identified as the Krt19+ Nkcc1+ population, were located farther from acinar cells. A human fetal lacrimal gland transcriptomic study showed expression of myoepithelial markers at a time when acinar cells acquire secretory functions (Farmer et al., 2017). With time, the KRT5+ KRT14+ cell population (myoepithelial and basal ductal markers) increased in both human and mouse glands. If similar progress occurs in the organoid development from expansion to maturation, it needs to be studied. Based on published studies, acinar (MIST1, AQP5), ductal (LCN2, WFDC2, CK5), and myoepithelial (ACTA2, CK14, CNN3, MYL9) markers can be used in future organoid studies (Bannier-Hélaoüet et al., 2021; Farmer et al., 2017; Asal et al., 2023).

Epithelial polarity has not been evaluated in the published studies. In organoids, AQP5 expression in the cytoplasm, without the characteristic luminal-side expression observed in three studies (as seen in adult gland tissue), does not support the functional organization of the organoids as in tissue (Asal et al., 2023). Jeong et al. showed only luminal expression of AQP5, similar to that observed in acinar cells of glandular tissue. The expression of Na+ /K+ ATPase on both the luminal and basal sides, and of LCN2 towards the luminal side, might indicate some polarity in the cellular arrangement in their study. However, it has not been replicated across the studies. Cell-cell and cell-extracellular matrix interactions are essential for maintaining acinar cell polarity. Lamini-1, desmosomes between the apical side of acinar epithelial cells and hemidesmosomes between the basal side and the basement membrane have been postulated to drive polarity in mammary tissue (Bissell and Bilder, 2003). The factors that determine the polarity of lacrimal gland acinar cells in vivo remain unknown and warrant further study. Transmission electron microscopy studies are needed to determine whether cells secrete apically or basally. Only one study (Jeong et al., 2021) has reported TEM of organoids, in which the presence of dark, round cytoplasmic bodies suggestive of secretory granules indicated the acinar nature of the cells. However, the authors did not provide a detailed description of the diifferent cell populations or of the SMA-positive cells shown arranged around EpCAM-positive cells. The available figures did not show lumen formation within the organoid; instead, they showed a clump of cells. Hence, differentiation between the luminal and basal sides was not possible. Studies have quantified proteins in culture supernatants or performed stimulatory experiments with neurotransmitters, but they cannot establish polarity.

### 3.6 Functional assays

To assess the secretory functions of the generated organoids, they were exposed to different stimulants, including pilocarpine, forskolin, carbachol, dbcAMP, Norepinephrine, and VIP to varying concentrations across studies. Asal et al. (2023) stimulated organoids with carbachol and forskolin, observing increased calcium levels and N-acetylglucosa-minidase (NAG) secretion, an indicator of lysozyme activity. Prolonged stimulation also led to a noticeable increase in organoid size. Jeong et al. (2021) used pilocarpine, which increased calcium concentration and β-hexosaminidase secretion. Bannier-Hélaoüet et al. (2023) applied forskolin and norepinephrine to induce organoid swelling, signaling water secretion, while their earlier study (2021) showed forskolin, norepinephrine, and pilocarpine treatment caused a measurable increase in organoid size, suggesting responsiveness to neurotransmitter-induced water secretion. Forskolin and dbcAMP increased the organoid diameter by 50% in 4 h, while pilocarpine and norepinephrine/VIP stimulation caused 25% and 50% swelling, respectively. The expression of chloride (CFTR, CLCN3, SLC12A4) and potassium ion channel (LRRC26) within the organoids, other than neurotransmitter receptor expression, supports the secretory potential of organoids (Bannier-Hélaoüet et al., 2021). However, there are few differences in their expression from the lacrimal gland tissue, such as muscarinic receptor (CHRM1 (in tissue) vs. CHRM3 (in organoids), and adrenergic receptor (ADRB1, ADRA1A (very low in tissue) vs. ADRB1(in organoids). Hayashi et al. (2022) found out forskolin-induced swelling, with approx. 30% increase in area after 16 h, indicating ion transport capabilities. Rodboon et al., 2022a,b reported that carbachol enhanced calcium levels and epithelial polarization, with a twofold increase in trans-epithelial electrical resistance (TEER), confirming organoid responsiveness to parasympathetic stimulation.

In addition to the swelling assays, two studies evaluated proteomics and metabolomics. Jeong et al. (2021) identified 776 proteins in the culture medium after pilocarpine treatment. There was a significant upregulation of 66 differentially expressed proteins (DEPs), predominantly linked to exosomes, suggesting possible tear production. Asal et al. (2023) identified 127 metabolites in cells and 178 in culture media, highlighting a metabolic shift from amino acid to lipid metabolism as the cells differentiated upon forskolin stimulation, indicating the acquisition of lacrimal functional phenotype.

### 3.7 Organoid transplantation in an animal model

Four studies reported the engraftment of organoids in mice or rats. The studies used various methods of injecting lacrimal gland organoids: Jeong et al. (2021) used cell clumps injected into the extra-orbital space, while Bannier-Hélaoüet et al. (2021); Bannier-Hélaoüet et al. (2023) injected organoids directly into the NOD-SCID mouse lacrimal gland, either as individual units or in a suspension. Hayashi et al. (2022) placed cultured organoids into the rat’s lacrimal gland tissue. The post-transplantation studies evaluated organoid integration and functionality over one week to two months. Jeong et al. (2021) demonstrated that GFP-labeled lacrimal gland organoids successfully engrafted into dry eye disease mouse model. After 14 days, GFP signals and the water channel protein AQP5 were detected, indicating the regenerative potential of the organoids. The lacrimal-gland-like organoids were transplanted into rats with partial gland removal by Hayashi et al. (2022). The following day, human organoid material was identified, and by four weeks, ductal structures were observed along with expression of markers such as KRT14, PAX6, CDH1, and AQP5. Bannier-Hélaoüet et al. (2021) found that the transplanted organoids formed duct-like structures by two weeks, remained viable for two months, and expressed tear-related proteins Lipocalin 2, Lactoferrin, and Lysozyme. Bannier-Hélaoüet et al. (2023) confirmed the engraftment of the organoid using the human nucleolar antigen staining. Nguyen et al. (2025) explored the role of WNT mimetics in promoting acinar differentiation in the lacrimal gland organoids. Lacrimal gland function was restored when they administered a WNT mimetic (L-F127) locally in a duct-ligation-induced dry eye mouse model that increased tear secretion starting from day 7.

## 4 Discussion

The three essential characteristics of a lacrimal gland organoid should be the spatial arrangement of acinar, myoepithelial, and ductal cells, lumen formation, and functional secretory capability. Lacrimal gland organoids generated from iPSCs and human or mouse tissue biopsies replicated some structural and functional aspects of the lacrimal gland, but not completely. The secretory function of the organoids was confirmed by the expression of tear proteins, including aquaporin 5, lactoferrin, and lysozyme, using stimulation and molecular analyses. Although protocols varied across studies, the organoids demonstrated calcium influx, N-acetyl-β-glucosaminidase activity, and swelling, confirming their secretory functions. The cellular composition of the organoids varied, including acinar, ductal epithelial, mesenchymal, and myoepithelial-like cells. RNA sequencing and PAX6 knockout studies highlighted the cellular diversity and gene-specific role of PAX6 (Makarenkova et al., 2000) in organoids. Lumen formation was noted in two studies (Bannier-Hélaoüet et al., 2021; Hayashi et al., 2022), whereas one study reported the lumen in the initial developmental stages (Asal et al., 2023). The successful engraftment of the organoids in animal models, studied for 2 to 8 weeks, revealed their functional capacity and ability to secrete proteins. Future studies with longer periods of engraftment and the study of changes on the ocular surface following organoid transplantation are needed to validate the positive effects of lacrimal gland organoids.

Lacrimal gland organoids have been developed from gland tissue biopsy (n = 4) without dedifferentiating into iPSCs, and some have used human iPSCs to create gland organoids (n = 2). For gland tissue biopsies, the digestion protocol shows little variation across studies; all used collagenase, but the use of dispase and ROCK inhibitors varied. Once the gland was digested, Matrigel was used to culture the cells. Again, there was variation in the culture medium used in studies, as highlighted in Table 1. With differing media composition and tissue sources, the average time for iPSCs to fully differentiate into lacrimal gland organoids ranged from 7 to 10 weeks. In studies in which tissue biopsy was the source of organoid development, the time required was 9-10 days in one study (Bannier-Hélaoüet et al., 2021) and 7-14 days in another (Bannier-Hélaoüet et al., 2023). Once the organoids were formed, they could be passaged for 19 to 40 passages. Lacrimal gland organoids development requires standardization/optimization for long-term culture to preserve progenitor cells and native lacrimal gland molecular expression. It is unclear if the organoids’ molecular signature/gene expression changed with passages or if any differences exist between early and late passages. Also, it has not been studied whether the differences in the media composition would affect the passaging duration. However, in the study using FGF10 in the media, the organoids could grow up to 40 passages (Bannier-Hélaoüet et al., 2021), highlighting the role of FGF10 in driving epithelial cell proliferation, branching morphogenesis, and differentiation necessary for gland formation (Makarenkova et al., 2000). Future studies can design the work to solve the above unknown factors in lacrimal gland organoids.

Lacrimal gland organoids were developed to have an artificial lab-grown lacrimal gland. Hence, the ideal organoid should match the lacrimal gland’s different cell types, molecular expression, and secretory function. Of six studies, epithelial markers were confirmed using either of molecular techniques (Table 4) in five studies, four studies have tested for mesenchymal markers (Hayashi et al., 2022; Asal et al., 2023; Jeong et al., 2021; Bannier-Hélaoüet et al., 2021) and only three studies have looked at developmental markers (Hayashi et al., 2022; Asal et al., 2023; Bannier-Hélaoüet et al., 2021), such as PAX6, SOX, OCT. Gene expression studied in two studies has shown the presence of acinar, ductal-like, and myoepithelial cells in the organoids (Bannier-Hélaoüet et al., 2021; Bannier-Hélaoüet et al., 2023). One of the significant issues in lacrimal gland culturing has been the inability to grow both acinar and ductal epithelial cells, as these two populations share a similar secretory function and epithelial nature. One study showed the presence of ductal-like cells, as confirmed by LCN2 and WFDC2 gene expression, using single-cell sequencing of the human lacrimal gland (Bannier-Hélaoüet et al., 2021). However, the population of these ductal-like cells was significantly lower than that of acinar epithelial or stromal cells. Another essential cell type within the lacrimal gland is the myoepithelial cell, which is responsible for the contraction of acinar epithelial cells. Myoepithelial cells’ presence in the lacrimal gland organoids was shown in five studies. The used marker was α-SMA (Hayashi et al., 2022; Asal et al., 2023; Bannier-Hélaoüet et al., 2021). However, one study (Bannier-Hélaoüet et al., 2021) showed the presence of other markers, such as ACTA2, KRT5, MYH11, MYLK, and TPM2, using gene expression. Two studies (Hayashi et al., 2022; Rodboon et al., 2022a,b) demonstrated the presence of myoepithelial-like/ductal progenitor cells (KRT14 positive) during organoid development. These cells are essential for stimulating the secretion of intracytoplasmic contents into the gland ducts. The structural organization of cells within the organoids did not match that of the human lacrimal gland, with acinar cells surrounded by myoepithelial cells. However, scRNA-seq revealed the presence of myoepithelial cell populations.

### 4.1 Immune cells and lacrimal gland organoid

The potential therapeutic applications of the lacrimal gland organoid include understanding the mechanisms of gland damage in dry eye disease and its translational use for regenerating the gland. The major drawback is the absence of an adaptive immune system, which makes it challenging to study immune-mediated lacrimal gland damage, which is commonly observed in aqueous-deficient dry eyes. One possible way is to co-culture immune cells with gland organoids in vitro using 3D-printed frameworks that enable constant flow, as in a bioreactor. In their current state, organoids are not suitable models for studying dry eye disease. However, their potential to mimic the native glandular architecture, containing acinar cells, myoepithelial cells, and mesenchymal cells, is advantageous compared with gland spheroids or 2D culture techniques. Further in vitro studies are needed to explore them as an in vitro model of the lacrimal gland. For translational use, these organoids must function in the host and exhibit a profile similar to that in culture. All studies have shown a positive secretory response of lacrimal gland organoids upon stimulation with various stimulants, such as pilocarpine and forskolin, in culture media. However, their effects on tear levels in animal models have not been studied. Only four studies have tested the organoid’s survival and function in the mouse or rat model (Hayashi et al., 2022; Jeong et al., 2021; Bannier-Hélaoüet et al., 2021; Bannier-Hélaoüet et al., 2023). The organoids were viable for 8 weeks post-transplantation and successfully engrafted into the host lacrimal gland. Only two studies used severe combined immunodeficiency mice, and both injected organoids derived from human sources into the mice without systemic immunosuppression. Histological analysis did not report any immune cell infiltration in these studies; hence, it is unclear whether these organoids would elicit a host inflammatory response after cross-species transplantation. The secretory function of these organoids was confirmed based on the expression of lactoferrin and lysozyme rather than tear volume or change in the ocular surface status in DED models. Future studies should examine the effects of lacrimal gland organoid supplementation on the animal model’s ocular surface and tear film. Also, integrating the host neural and vascular system into transplanted organoids is essential for their long-term effect and survival and needs further study.

### 4.2 Lacrimal gland and other exocrine glands

Salivary glands and the lacrimal gland share a similar acinar-ductal organization. Salivary gland transplantation in severe dry eye disease has been reported to restore tear production, though corneal edema can occur due to the hypo-osmolar tear film (Singh et al., 2022). Numerous studies have been published on salivary gland organoids. Recent advances in lacrimal gland organoid research have demonstrated similarities to organoids derived from other exocrine glands, such as the salivary gland (Zhao et al., 2021; Yoon et al., 2024) and the pancreas (Grapin-Botton and Kim, 2022; Broutier et al., 2016). There are similarities and differences in the development and expression of salivary and lacrimal gland organoids. Lacrimal and salivary gland organoids replicate essential aspects of glandular architecture, including acinar-like structures mimicking native glandular secretion (Hayashi et al., 2022; Asal et al., 2023; Jeong et al., 2021; Bannier-Hélaoüet et al., 2021; Bannier-Hélaoüet et al., 2023). However, lacrimal and salivary gland organoids differ in cell composition, secretion patterns, and development. In salivary gland organoids, key signaling pathways such as Wnt and Notch have been identified as critical cellular differentiation regulators and glandular identity maintenance (Yoon et al., 2024; Dang et al., 2009). These pathways also play a role in the lacrimal gland organoids (Bannier-Hélaoüet et al., 2023). Additionally, the cellular composition of lacrimal gland organoids may differ from that of salivary gland organoids. Salivary gland organoids typically contain a diverse population of acinar, ductal, and myoepithelial cells, reflecting the complexity of the salivary gland tissue (Yoon et al., 2024; Dang et al., 2009; Kim et al., 2021). However, studies of lacrimal gland organoids indicate a predominance of acinar-like cells with less contribution of myoepithelial cells. This suggests a potential difference in the cellular architecture and these cell types’ functional roles in the respective glands (Hayashi et al., 2022; Asal et al., 2023; Jeong et al., 2021; Bannier-Hélaoüet et al., 2021). Another difference was in lumen formation. Adding VIP and/or retinoic acid to the submandibular gland organoid increased the lumen formation via VIPR1 (Kim et al., 2021). VIPR1 was also expressed in lacrimal gland organoids (Bannier-Hélaoüet et al., 2021). However, its effects on the lumen formation were not tested. In addition to VIP, retinoic acid synergized with VIP for lumen formation in SMG organoids. These factors can be explored in lacrimal gland organoids. Like lacrimal gland organoids, FGF10 is essential for developing salivary gland organoids, especially in branching (Sui et al., 2020). This knowledge can help us use organoid technology to create therapies for restoring lacrimal gland function in conditions like Sjögren’s syndrome and other tear-production disorders.

**Fig. 3:**
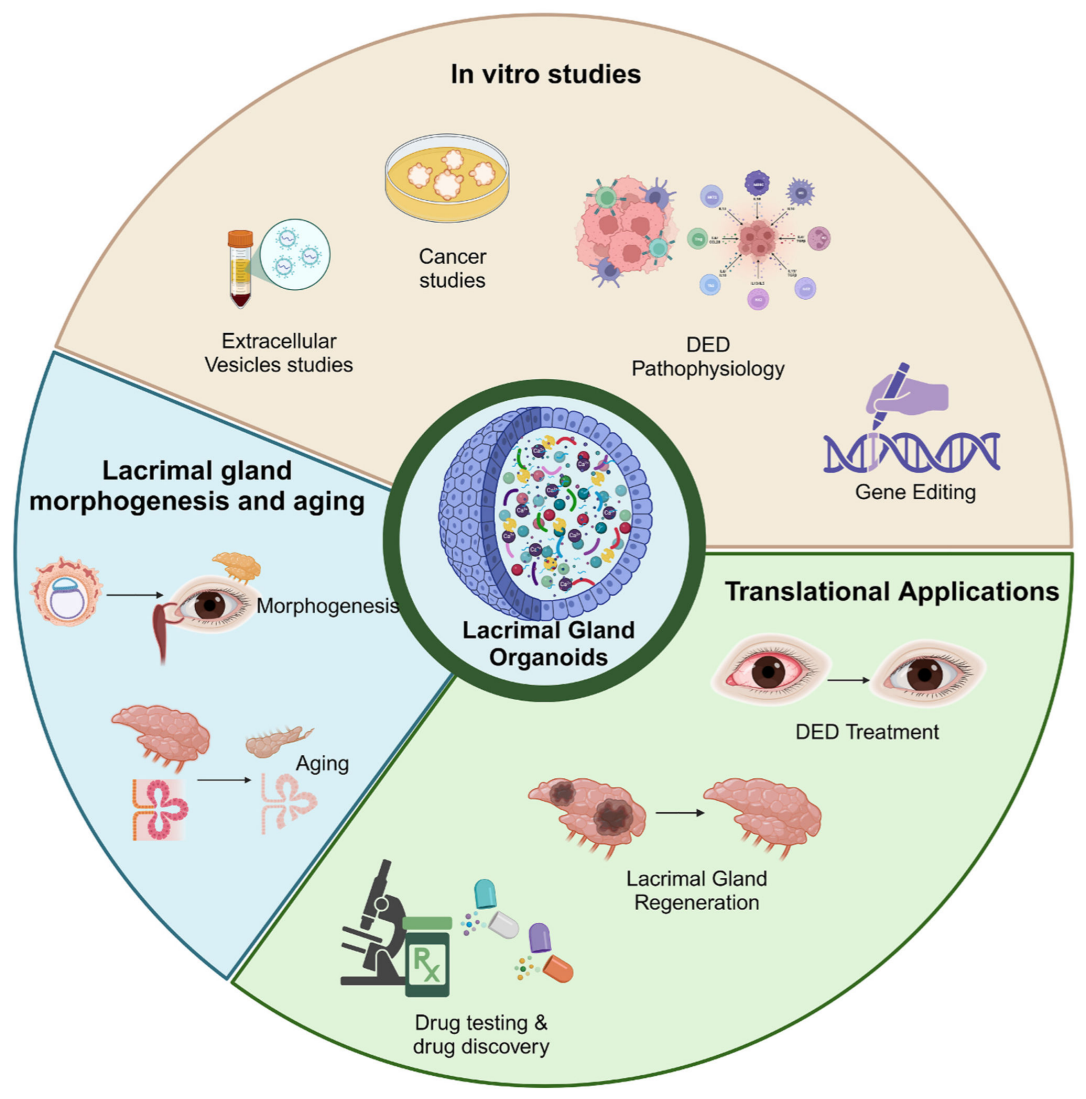
Schematic showing different possible applications of lacrimal gland organoids.

Compared with 2D cell cultures, lacrimal gland organoids more effectively recapitulate in vivo tissue complexity and cell-cell interactions. However, no direct comparison studies exist on the cell populations obtained in lacrimal gland 2D cultures versus gland organoids. In a 3D organoid environment, epithelial cells differentiate into acinar and ductal lineages, developing a lobular architecture with branching morphogenesis similar to native lacrimal gland tissue (Garg and Zhang, 2017). Additionally, organoids had a lumen and retained myoepithelial cells around acinar clusters, whereas 2D cultures lack proper lacrimal gland function, such as branching and secretion. This branching is critical for the formation of acinar units specialized for tear production (Garg and Zhang, 2017). Fig. 3 depicts the possible translational applications of lacrimal gland organoids. Lacrimal gland organoids exhibit significant translational potential for developing therapies for dry eye disease, such as Sjögren’s syndrome, by providing a platform for personalized medicine and regenerative approaches (Abdal et al., 2025). One such application is the use of lacrimal gland organoids to study the senescence pathway in acinar cells, where senescence was reversed with HMGB1-BoxA gene therapy (Ferreira et al., 2024). Another application was determining the role of Pax6 in the lacrimal gland morphogenesis. Two studies (Bannier-Hélaoüet et al., 2021, 2023) generated Pax6 knockouts. Pax6 knockout (KO) organoids exhibited reduced proliferation, downregulation of genes essential for lacrimal gland function (e.g., Chrm1, Aqp5), and an increased interferon response, indicating stress-induced immune activation.

The ability to recapitulate structural and functional characteristics of native organs, including secretory functions, makes them valuable for testing patient-specific drug responses. However, limitations exist, challenges in maintaining long-term viability and secretory capacity, and a predominance of acinar-like cells with less emphasis on myoepithelial components. Addressing these challenges is essential for enhancing the clinical applicability of lacrimal gland organoids in regenerative medicine. Organoids are a key source for studying gland morphogenesis. A study of transcriptomics over short-term vs long-term cultured organoids would help understand the key differentiation markers.

In conclusion, lacrimal gland organoids generated from iPSCs and tissue biopsies partially recapitulate the structural and functional characteristics of native lacrimal glands, demonstrating key expression of tear proteins and secretory function. The cellular composition still has not matched that of the lacrimal gland, including acinar, ductal, mesenchymal, and myoepithelial-like cells, which vary across studies and warrant further investigation. While successful engraftment studies show post-sacrifice assessment over 2 to 8 weeks, long-term assessments are necessary to validate prolonged integration and tear volume levels. Despite their translational potential for treating conditions like Sjögren’s syndrome, challenges related to long-term viability and differences in cellular composition must be addressed to enhance their clinical applicability in regenerative medicine.

## Figures and Tables

**Table 1:** Method and media composition used for generation of lacrimal gland organoids across different studies.

Author		Source		Digestion/ Differentiation Method		Matrix		Media			Duration/ Passage		Key Outcome
								Expansion	Differentiation				
Hayashi et al. (2022)		The hiPS cell line 201B7 and human ES cell line (KhES-1)		SEAM method for differentiating hPS cells into ocular cell lineages^44^		Laminin-511 E8 fragments, Matrigel		Culture in StemFit medium for 10 days. **Differentiation Medium: **Serum-free DM (GMEM) with 10% knockout serum replacement, 1 mM sodium pyruvate, 0.1 mM non-essential amino acids, 2 mM l-glutamine, 100 U/ml penicillin potassium, 100 μg/ml streptomycin sulfate, and 55 μM monothioglycerol **Epithelial differentiation: **After 4 weeks, switch to EDM (DM and CnT-PR without EGF and FGF2) with 20 ng/ml KGF, 10 μM Y-27632, 100 U/ml penicillin potassium, and 100 μg/ml streptomycin sulfate **Ocular Surface Epithelial Differentiation Medium (OSEM)**: Replace with OSEM (DMEM/F12 with 2% B-27 supplement, 20 ng/ml KGF, 10 μM Y-27632, 100 U/ml penicillin potassium, 100 μg/ml streptomycin sulfate) for 0 to 6 additional weeks. **Lacrimal Gland Culture Medium: **DMEM/F12 containing 2% B-27 supplement, 10 ng/ml EGF, 10 μM Y-27632, 100 U/ml penicillin potassium, 100 μg/ml streptomycin sulfate			6 weeks		3D acinar-like structures
Asal et al. (2023)		hiPSC Lines		Multi-zonal differentiation Method^45^		Matrigel		mTeSR1 medium for hiPSCs Eye Field Differentiation Medium-DMEM/F12 and Neurobasal Medium supplemented with 2 mM L-GlutaMAX, 0.1 mM non-essential amino acids, 0.1 mM monothioglycerol, 1% N2 MAX supplement ocular cell differentiation (OD) medium composed of DMEM/F12 supplemented with 10% knockout serum replacement 2 mM L-GlutaMAX, 0.1 mM NEAA, and 0.1 mM **monothioglycerol**			7 weeks		Branching morphogenesis
Jeong et al. (2021)		Human and Mouse LG Tissue		0.125 mg/mL dispase II 0.1 mg/mL DNase I 0.125 mg/ml collagenase II		Matrigel		Salivary Gland Organoid Media supplemented with 10 mM nicotinamide, 500 nM A83, and 100 ng/mL noggin			19 passages		Long-term growth
Bannier-Hélaouët et al. (2021)		Human and Mouse LG Tissue		1 mg/mL collagenase and 10 mM ROCK inhibitor Y-27632 in AdDMEM/F12 while shaking		Cultrex Path clear Reduced Growth Factor Basement Membrane Extract (BME)		**Mouse:** AdDMEM/F12 supplemented with B27, Glutamax, HEPES, 100 U/ mL Pen/Strep, 100 μg/mL Primocin, **1.25 mM N-acetylcysteine**, 10 mM nicotinamide, and growth factors: 1% Noggin conditioned medium, 1% R-spondin 3 conditioned medium, 0.5 μM A83-01, **1 μM PGE2**, 1 μM FSK, 100 ng/mL FGF10 **Human: **AdDMEM/F12 supplemented with B27, Glutamax, HEPES, 100 U/ mL Pen/Strep, 100 μg/mL Primocin, 1.25 mM **N-acetylcysteine**, and growth factors: 2% Noggin conditioned medium, 2% R-spondin 3 conditioned medium, 0.5 μM A83-01, 1 μM FSK, 100 ng/mL FGF10	**Mouse:** AdDMEM/F12 supplemented with B27, Glutamax, HEPES, 100 U/mL Pen/Strep, 100 μg/ mL Primocin, 1 μM FSK, 100 ng/mL FGF10 **Human: **AdDMEM/F12, Glutamax, HEPES, 100 U/mL Pen/Strep, 1.25 mM N-acetylcysteine, 10 μM DAPT		Mouse: 40p; Human: 20p		High viability/ expansion
Bannier-Hélaouët et al. (2023)		Human and Mouse LG Tissue		Base medium (DMEM/F12, Glutamax, HEPES and Penicillin/ Streptomycin), Collagenase I (20 mg/ml)		ECM		**Mouse:** Base Medium supplemented with R-spondin 3 conditioned medium, Noggin conditioned medium, B27 supplement, **500 mM N-acetylcysteine**, 1M Nicotinamide, 10 mM Forskolin, 30 mM A83-01, **10 mM Prostaglandin E2 (PGE2)**, 100 mM FGF10, 10 mM Y-27632 **Human: **Same as Mouse, excluding 10 mM Prostaglandin E2 (PGE2)	**Mouse:** Base Medium supplemented B27 supplement, 10 mM Forskolin, 100 mM FGF10, 10 mM Y-27632 **Human: **Base Medium supplemented with 500 mM N-acetylcysteine and 10 mM DAPT		7-10days expansion; 5 days differentiation		Rapid proliferation
Rodboon et al. (2022)		Porcine LG Tissue		Magnetic three-dimensional bioprinting (M3DB)^46^		Reduced Growth Factor Basement Membrane Extract (BME)		Epithelial Enrichment Media (serum-free DKSFM supplemented with EGF, FGF-7 and FGF-10)			8 days		Size: 173 ± 17.6 → 628 ± 24.3 pm
Nguyen et al. (2025)		Mouse lacrimal gland tissue		Collagenase, hyaluronidase, DNase digestion		Matrigel		Advanced DMEM/F12 + B27, N2, EGF, FGF2, FGF10 + antibody-based WNT mimetic (FZD1/2/7 ± RSPO)			Multi-passage expansion		WNT-driven acinar organoid expansion

**Table 2:** Role of different components of the culture medium used for organoids.

Sr. No.	Component	Role in lacrimal gland organoid development
1	EGF (Epidermal Growth Factor)	Promotes epithelial cell proliferation, survival, and differentiation; supports epithelial integrity.
2	FGF2 (Fibroblast Growth Factor 2)	Enhances proliferation, stem cell maintenance, and epithelial-mesenchymal interactions; prevents apoptosis.
3	KGF (Keratinocyte Growth Factor/FGF7)	Stimulates epithelial cell proliferation; supports differentiation of acinar and ductal structures.
4	Y-27632	ROCK inhibitor that prevents apoptosis in single-cell cultures; improves cell survival during passaging.
5	B-27 Supplement	Provides essential vitamins, antioxidants, and growth factors; supports cell survival, growth, and differentiation.
6	L-GlutaMAX	Stabilized form of L-glutamine that enhances cell metabolism and prevents ammonia toxicity.
7	Monothioglycerol	Antioxidant that protects cells from oxidative stress during organoid growth.
8	Nicotinamide	Promotes epithelial differentiation and supports cell survival; enhances acinar cell formation.
9	A83	TGF-p receptor inhibitor that suppresses unwanted fibroblast activity and promotes epithelial cell expansion.
10	Noggin	BMP inhibitor that maintains stemness and promotes epithelial growth and branching morphogenesis.
11	Primocin	Broad-spectrum antibiotic used to prevent bacterial, mycoplasma, and fungal contamination.
12	N-Acetylcysteine (NAC)	Antioxidant that reduces oxidative stress and enhances cell survival.
13	R-Spondin 3	Wnt pathway activator that promotes stem cell maintenance, epithelial proliferation, and tissue regeneration.
14	PGE2 (Prostaglandin E2)	Enhances cell proliferation, survival, and tissue regeneration; supports epithelial growth.
15	FSK (Forskolin)	Activates cyclic AMP (cAMP) signaling, enhancing fluid secretion and promoting organoid swelling (useful for assessing gland function).
16	FGF10	Crucial for branching morphogenesis and acinar differentiation; enhances epithelial proliferation.
17	DAPT	Y-secretase inhibitor that blocks Notch signaling, promoting differentiation of acinar and ductal cell types.
18	WNT mimetics (antibody-based)	Direct activation of canonical WNT signaling to maintain acinar identity and proliferation of acinar-derived lacrimal gland organoids

**Table 3:** Key developmental signaling pathways converging on lacrimal gland organoid formation.

Signaling component	Developmental pathway	Functional role in organoid development	Studies
FGF10	FGF signaling	Central inductive cue driving epithelial budding, branching morphogenesis via FGFR2b	Hayashi et al. (2022); Asal et al. (2023); Jeong et al. (2021); Bannier-Hélaouët et al. (2021); Rodboon et al., 2022a,b
Noggin	BMP signaling (inhibition)	Indirect modulation of epithelial differentiation by suppressing BMP activity and maintaining epithelial progenitor states	Jeong et al. (2021); Bannier-Hélaouët et al. (2021)
R-spondin 3	Wnt signaling (activation)	Potentiation of canonical Wnt signaling to sustain epithelial progenitor expansion during early organoid culture	Bannier-Hélaouët et al. (2021)
DAPT	Notch signaling (inhibition)	γ-secretase inhibition to modulate epithelial differentiation during later stages of organoid maturation	Bannier-Hélaouët et al. (2021)

**Table 4:** Biomarkers used for identifying the cell populations within lacrimal gland organoids.

Study	Technique	Developmental Markers			Epithelial Markers			Mesenchymal Markers			Proliferation Markers	
		Marker	Day		Marker	Day		Marker	Day		Marker	Day
Hayashi et al. (2022)	**IF/IHC**	PAX6, TP63, BARX2, CHX10	2-14 weeks		AQP5, E-Cadherin, Lactoferrin, Histatin1, KRT12, KRT13, KRT14, KRT15, KRT19, CD44, SOX9, MUC16, CD31	2-14 weeks		α-SMA, Calponin	2-14 weeks		YAP1	-
	**Flow Cytometry**	-	-		SSEA-4, CD200, ITGB4	10-11 weeks		-	-		-	-
	**Westen Blot**	BARX2	-		-	-		-	-		-	-
	**qRT-PCR**	PAX6, BARX2, SIX1, SIX2, SOX17, GATA4, TP63, RUNX1, FGFR1 (IIIb), FGFR2(IIIb)	2-14 weeks		AQP5, Lactoferrin, Lysozyme, KRT12, KRT13, KRT14, KRT15, N-Cadherin, CD44, E-Cadherin, SOX9, Histatin1	10 weeks		α-SMA, Calponin, CTGF	2-14 weeks		-	-
	**ELISA**	Lactoferrin, Lysozyme			-			-			-	-
Asal et al. (2023)	**IF/IHC**	PAX6, TP63, OCT3/4, NANOG, SOX2, PITX2, FOXC1, SOX10	Day 21		AQP5, Lysozyme, KRT5, Lactoferrin, Lipocalin-2, KRT13, KRT14, KRT19, Claudin 1	Day 21		α-SMA, Calponin	Day 30 and 35		-	-
	**Flow Cytometry**	-	-		EpCAM, SSEA-4, NGFR, TRA-1-60	Day 21 for EpCAM and rest for Pluripote-ncy of the cells		-	-		-	-
	**qRT-PCR**	PAX6, TP63, FOXC1, LMX1B, FGF10, OTX1, BMP7, SOX10	Day 21		AQP5, Lactoferrin, KRT13, KRT15, NGFR	Day 21		-	-		-	-
Jeong et al. (2021)	**IF/IHC**	-	-		AQP5, E-Cadherin, Lysozyme,	Day 4		Vimentin, α-SMA	Day 4		Ki67	Day 4
	**Flow Cytometry**	-	-		EpCAM	Day 4		-	-		-	-
	**qRT-PCR**	-	-		AQP5	Day 4		-	-		-	-
Bannier-Hélaouët et al. (2021)	**IF/IHC**	PAX6, TP63	>10 days		AQP5, Lysozyme, KRT5, Lactoferrin, WFDC2, SCGB2A1, BPIFA1, Histatin1, Lipocalin-2, PRR27	>10 days		α-SMA, Phalloidin-Atto 647N	>10 days		Ki67	>10 days
	**Western** **Blot**	PAX6	>10 days		-	-		-	-		-	-
	**qRT-PCR**	LGR5	>10 days		AQP5, KRT5, Lactoferrin, Lysozyme, WFDC2, CHRM1, CHRM3, SFTPD, Lipocalin-2, ADRB1, TNFRSF19, N-Cadherin, CD44, E-Cadherin, SOX9, Histatin1	>10 days		α-SMA, SERPINB2, SERPINE2	>10 days		-	-
Nguyen et al. (2025)	**IF/IHC**	-	-		BHLHA15 (MIST1), E-Cadherin	-		-	-		Ki67	-
	**qRT-PCR**	AXIN2, LGR5	-		BHLHA15 (MIST1), KRT7	-		-	-		-	-

## Data Availability

No data was used for the research described in the article.

## References

[R1] Abdal Dayem A, Bin Jang S, Lim N (2025). Advances in lacrimal gland organoid development: techniques and therapeutic applications. Biomed Pharmacother.

[R2] Asal M, Koçak G, Sari V (2023). Development of lacrimal gland organoids from iPSC derived multizonal ocular cells. Front Cell Dev Biol.

[R3] Bannier-Hélaoüet M, Geurts MH, Korving J, Begthel H, Clevers H (2023). Establishment, maintenance, differentiation, genetic manipulation, and transplantation of mouse and human lacrimal gland organoids. J Vis Exp.

[R4] Bannier-Hélaoüet M, Korving J, Ma Z (2024). Human conjunctiva organoids to study ocular surface homeostasis and disease. Cell Stem Cell.

[R5] Bannier-Hélaoüet M, Post Y, Korving J (2021). Exploring the human lacrimal gland using organoids and single-cell sequencing. Cell Stem Cell.

[R6] Bissell MJ, Bilder D (2003). Polarity determination in breast tissue: desmosomal adhesion, myoepithelial cells, and laminin 1. Breast Cancer Res.

[R7] Bron AJ, de Paiva CS, Chauhan SK (2019). TFOS DEWS II pathophysiology report. Ocul Surf.

[R8] Broutier L, Andersson-Rolf A, Hindley CJ (2016). Culture and establishment of self-renewing human and mouse adult liver and pancreas 3D organoids and their genetic manipulation. Nat Protoc.

[R9] Chakrabarty K, Shetty R, Ghosh A (2018). Corneal cell therapy: with iPSCs, it is no more a far-sight. Stem Cell Res Ther.

[R10] Chen X, Li S, Zhang Y, Ye L (2025). Progress and prospects in the treatment of lacrimal gland dysfunction diseases: from traditional treatment methods to stem cell and organoid therapies. Stem Cell Int.

[R11] Craig JP, Nichols KK, Akpek EK (2017). TFOS DEWS II definition and classification report. Ocul Surf.

[R12] Dang H, Lin AL, Zhang B, Zhang HM, Katz MS, Yeh CK (2009). Role for Notch signaling in salivary acinar cell growth and differentiation. Dev Dyn.

[R13] Dean C, Ito M, Makarenkova HP, Faber SC, Lang RA (2004). Bmp7 regulates branching morphogenesis of the lacrimal gland by promoting mesenchymal proliferation and condensation. Development (Cambridge, England).

[R14] Dean CH, Miller LA, Smith AN, Dufort D, Lang RA, Niswander LA (2005). Canonical Wnt signaling negatively regulates branching morphogenesis of the lung and lacrimal gland. Dev Biol.

[R15] Dietrich J, Massie I, Roth M, Geerling G, Mertsch S, Schrader S (2016). Development of causative treatment strategies for lacrimal Gland insufficiency by tissue engineering and cell therapy. Part 1: regeneration of lacrimal Gland tissue: can we stimulate lacrimal Gland renewal in vivo?. Curr Eye Res.

[R16] Donthineni PR, Doctor MB, Shanbhag S (2023). Aqueous-deficient dry eye disease: preferred practice pattern guidelines on clinical approach, diagnosis, and management. Indian J Ophthalmol.

[R17] Dvoriantchikova G, Tao W, Pappas S, Gaidosh G, Tse DT, Ivanov D, Pelaez D (2017). Molecular profiling of the developing lacrimal gland reveals putative role of notch signaling in branching morphogenesis. Investig Ophthalmol Vis Sci.

[R18] Erbani J, Aberdam D, Larghero J, Vanneaux V (2016). Pluripotent stem cells and other innovative strategies for the treatment of ocular surface diseases. Stem Cell Rev Rep.

[R19] Farmer DT, Nathan S, Finley JK, Shengyang Yu K, Emmerson E, Byrnes LE, Sneddon JB, McManus MT, Tward AD, Knox SM (2017). Defining epithelial cell dynamics and lineage relationships in the developing lacrimal gland. Development (Cambridge, England).

[R20] Ferreira JN, Bhummaphan N, Chaisuparat R, Van Phan T, Oo Y, Jaru-Ampornpan P, Matangkasombut O, Mutirangura A (2024). Unveiling senescence-associated ocular pathogenesis via lacrimal gland organoid magnetic bioassembly platform and HMGB1-Box A gene therapy. Sci Rep.

[R21] Finburgh EN, Mauduit O, Noguchi T (2023). Role of FGF10/FGFR2b signaling in homeostasis and regeneration of adult lacrimal gland and corneal epithelium proliferation. Investig Ophthalmol Vis Sci.

[R22] Foster JW, Wahlin K, Adams SM, Birk DE, Zack DJ, Chakravarti S (2017). Cornea organoids from human induced pluripotent stem cells. Sci Rep.

[R23] Garg A, Zhang X (2017). Lacrimal gland development: from signaling interactions to regenerative medicine. Dev Dyn.

[R24] Gleixner S, Zahn I, Dietrich J (2024). A new immortalized human lacrimal Gland cell line. Cells.

[R25] Grapin-Botton A, Kim YH (2022). Pancreas organoid models of development and regeneration. Development.

[R26] Govindarajan V, Ito M, Makarenkova HP, Lang RA, Overbeek PA (2000). Endogenous and ectopic gland induction by FGF-10. Dev Biol.

[R27] Han Y, Jiang L, Shi H (2021). Effectiveness of an ocular adhesive polyhedral oligomeric silsesquioxane hybrid thermo-responsive FK506 hydrogel in a murine model of dry eye. Bioact Mater.

[R28] Hann LE, Tatro JB, Sullivan DA (1989). Morphology and function of lacrimal gland acinar cells in primary culture. Investig Ophthalmol Vis Sci.

[R29] Hayashi R, Ishikawa Y, Sasamoto Y (2016). Co-ordinated ocular development from human iPS cells and recovery of corneal function. Nature.

[R30] Hayashi R, Okubo T, Kudo Y (2022). Generation of 3D lacrimal gland organoids from human pluripotent stem cells. Nature.

[R31] Higa K, Higuchi J, Kimoto R (2020). Human corneal limbal organoids maintaining limbal stem cell niche function. Stem Cell Res.

[R32] Hongisto H, Vattulainen M, Ilmarinen T, Mikhailova A, Skottman H (2018). Efficient and scalable directed differentiation of clinically compatible corneal limbal epithelial stem cells from human pluripotent stem cells. J Vis Exp.

[R33] Jeong SY, Choi WH, Jeon SG (2021). Establishment of functional epithelial organoids from human lacrimal glands. Stem Cell Res Ther.

[R34] Jones L, Downie LE, Korb D (2017). TFOS DEWS II management and therapy report. Ocul Surf.

[R35] Jones MR, Dilai S, Lingampally A (2019). A comprehensive analysis of fibroblast growth factor receptor 2b signaling on epithelial tip progenitor cells during early mouse lung branching morphogenesis. Front Genet.

[R36] Joshi VP, Singh S, Thacker M (2023). Newer approaches to dry eye therapy: nanotechnology, regenerative medicine, and tissue engineering. Indian J Ophthalmol.

[R37] Kasal K, Güven S, Utine CA (2022). Current methodology and cell sources for lacrimal gland tissue engineering. Exp Eye Res.

[R38] Kim D, Yoon YJ, Choi D, Kim J, Lim JY (2021). 3D organoid culture from adult salivary gland tissues as an ex vivo modeling of salivary gland morphogenesis. Front Cell Dev Biol.

[R39] Li Z, Duan H, Li W (2019). Rapid differentiation of multi-zone ocular cells from human induced pluripotent stem cells and generation of corneal epithelial and endothelial cells. Stem Cell Dev.

[R40] Makarenkova HP, Ito M, Govindarajan V (2000). FGF10 is an inducer and Pax6 a competence factor for lacrimal gland development. Development.

[R41] Manafi N, Shokri F, Achberger K (2021). Organoids and organ chips in ophthalmology. Ocul Surf.

[R42] Massie I, Spaniol K, Barbian A, Geerling G, Metzger M, Schrader S (2018). Development of lacrimal gland spheroids for lacrimal gland tissue regeneration. J Tissue Eng Regen Med.

[R43] Møller-Hansen M, Larsen AC, Wiencke AK (2024). Allogeneic mesenchymal stem cell therapy for dry eye disease in patients with Sjögren’s syndrome: a randomized clinical trial. Ocul Surf.

[R44] Nguyen DH, Beuerman RW, Halbert CL, Ma Q, Sun G (1999). Characterization of immortalized rabbit lacrimal gland epithelial cells. In Vitro Cell Dev Biol Anim.

[R45] Nguyen H, Fletcher RB, Lopez T, Whisler E, Logas KR, Dhaliwal N, Suen T, Zhang M, Dilip A, Yuan TZ, Go H (2025). WNT mimetic-induced lacrimal gland regeneration reverses aqueous tear deficiency. Transl Vis Sci Technol.

[R46] Oliver C (1980). Isolation and maintenance of differentiated exocrine gland acinar cells in vitro. In Vitro.

[R47] Pan Q, Angelina A, Marrone M, Stark WJ, Akpek EK (2017). Autologous serum eye drops for dry eye. Cochrane Database Syst Rev.

[R48] Pan Y, Carbe C, Powers A, Zhang EE, Esko JD, Grobe K, Feng GS, Zhang X (2008). Bud specific N-sulfation of heparan sulfate regulates Shp2-dependent FGF signaling during lacrimal gland induction. Development (Cambridge, England).

[R49] Patel N, Sharpe PT, Miletich I (2011). Coordination of epithelial branching and salivary gland lumen formation by Wnt and FGF signals. Dev Biol.

[R50] Qiu X, Yang J, Liu T, Jiang Y, Le Q, Lu Y (2012). Efficient generation of lens progenitor cells from cataract patient-specific induced pluripotent stem cells. PLoS One.

[R51] Ranjan A, Basu S, Singh S (2024). Punctal cautery in dry eye disease: a systematic review. Ocul Surf.

[R52] Rodboon T, Souza GR, Mutirangura A, Ferreira JN (2022a). Magnetic bioassembly platforms for establishing craniofacial exocrine gland organoids as aging in vitro models. PLoS One.

[R53] Rodboon T, Yodmuang S, Chaisuparat R, Ferreira JN (2022b). Development of high-throughput lacrimal gland organoid platforms for drug discovery in dry eye disease. SLAS Discov.

[R54] Schrader S, Kremling C, Klinger M, Laqua H, Geerling G (2009). Cultivation of lacrimal gland acinar cells in a microgravity environment. Br J Ophthalmol.

[R55] Singh S, Basu S, Geerling G (2022). Salivary gland transplantation for dry eye disease: indications, techniques, and outcomes. Ocul Surf.

[R56] Singh S, Basu S (2020). The human lacrimal gland: historical perspectives, current understanding, and recent advances. Curr Eye Res.

[R57] Singh S, Donthineni PR, Srivastav S, Jacobi C, Basu S, Paulsen F (2023). Lacrimal and meibomian gland evaluation in dry eye disease: a mini-review. Indian J Ophthalmol.

[R58] Singh S, Winter Z, Necker F (2024). New insights into lacrimal gland anatomy using 7T MRI and electron microscopy: relevance for lacrimal gland targeted therapies and bioengineering. Ocul Surf.

[R59] Sui Y, Zhang S, Li Y, Zhang X, Hu W, Feng Y, Xiong J, Zhang Y, Wei S (2020). Generation of functional salivary gland tissue from human submandibular gland stem/progenitor cells. Stem Cell Res Ther.

[R60] Tiwari S, Nair RM, Vamadevan P (2018). Establishing and characterizing lacrispheres from human lacrimal gland for potential clinical application. Graefes Arch Clin Exp Ophthalmol.

[R61] Veernala I, Jaffet J, Fried J (2022). Lacrimal gland regeneration: the unmet challenges and promise for dry eye therapy. Ocul Surf.

[R62] Williams DL, Mann BK (2014). Efficacy of a crosslinked hyaluronic acid-based hydrogel as a tear film supplement: a masked controlled study. PLoS One.

[R63] Yoon YJ, Kim D, Tak KY (2024). Salivary gland organoid culture maintains distinct glandular properties of murine and human major salivary glands. Nat Commun.

[R64] Yu MD, Park JK, Kossler AL (2021). Stimulating tear production: spotlight on neurostimulation. Clin Ophthalmol.

[R65] Zhang C, Du L, Pang K, Wu X (2017). Differentiation of human embryonic stem cells into corneal epithelial progenitor cells under defined conditions. PLoS One.

[R66] Zhao C, Meng C, Cui N, Sha J, Sun L, Zhu D (2021). Organoid models for salivary gland biology and regenerative medicine. Stem Cell Int.

